# Cytology, High-Risk HPV Genotyping, and Histology in Cervical Pathology: A Retrospective Cohort Study

**DOI:** 10.3390/diagnostics16193204

**Published:** 2026-10-02

**Authors:** Maria-Daniela Vasieș (Mazilu), Elena-Lavinia Rusu, Jasmina-Sorina Chiriac, Alexandra-Corina Faur, Elena-Rodica Heredea, Veronica-Daniela Chiriac

**Affiliations:** 1 Doctoral School of Medicine, Faculty of Medicine, “Victor Babeș” University of Medicine and Pharmacy, Eftimie Murgu Square No. 2, 300041 Timișoara, Romania; maria.vasies@umft.ro (M.-D.V.);; 2 Department I of Anatomy and Embryology, Faculty of Medicine, “Victor Babeș” University of Medicine and Pharmacy, Eftimie Murgu Square No. 2, 300041 Timișoara, Romania; faur.alexandra@umft.ro; 3 Department of Clinical Practical Skills, “Victor Babeș” University of Medicine and Pharmacy, Eftimie Murgu Square No. 2, 300041 Timișoara, Romania; 4 Department XII, Obstetrics-Gynecology I, Faculty of Medicine, “Victor Babeș” University of Medicine and Pharmacy, Eftimie Murgu Square No. 2, 300041 Timișoara, Romania

**Keywords:** papillomavirus infections, uterine cervical neoplasms, Papanicolaou test, cervical intraepithelial neoplasia, colposcopy

## Abstract

**Background/Objectives:** Cervical cancer prevention relies on cytology, high-risk human papillomavirus (HR-HPV) testing, and histological confirmation, but these modalities may disagree. We quantified test concordance and determinants of CIN2+ in a specialized gynecologic referral cohort with very low HPV vaccination coverage. **Methods:** We retrospectively analyzed 172 women evaluated for suspected or established cervical pathology between 2022 and 2024. Of these, 160 had a definitive HPV result and 130 had histology; histology-based performance analyses used complete cases and are conditional on biopsy verification. Cytology was categorized by the Bethesda system, HR-HPV genotypes were recorded, and histology was dichotomized as CIN2+ versus ≤CIN1. **Results:** Among 160 women with definitive HPV results, 109 (68.1%) were HPV-positive. HPV-positive women were younger (median 33 vs. 43 years, *p* < 0.001) and, among biopsied women, more frequently had CIN2+ (70.2% vs. 22.2%, *p* < 0.001). Cyto-histological agreement was fair (65.0%, κ = 0.32). HPV16/18 was strongly associated with CIN2+ (OR 6.85, 95% CI 2.54–18.49). Within histologically verified complete cases, HPV DNA positivity had the highest apparent sensitivity (91.7%), whereas HPV16/18 had the highest PPV (86.0%). **Conclusions:** Cytology and HR-HPV testing provide complementary information in this referral-enriched cohort. Because disease prevalence was high and biopsy verification was selective, predictive values and apparent diagnostic-performance estimates should not be extrapolated directly to population screening; external validation in primary-screening populations is required.

## 1. Introduction

Cervical cancer remains the fourth-most common malignancy among women worldwide and a leading cause of cancer death in low-resource and inadequately screened populations [1,2]. Virtually all cervical carcinomas are caused by persistent infection with high-risk genotypes of the human papillomavirus (HPV), a causal relationship that is stronger and more consistent than for almost any other human cancer [3,4]. This etiological clarity has transformed cervical cancer into a largely preventable disease, achievable through vaccination against oncogenic HPV types and organized screening able to detect and treat precursor lesions before invasion occurs; the World Health Organization has accordingly set targets for its global elimination [5]. Nonetheless, in several Central and Eastern European countries, including Romania, incidence and mortality remain among the highest in the European Union, reflecting low vaccination uptake and fragmented, opportunistic screening.

The natural history of HPV-induced cervical disease provides the biological rationale for screening. Most incident HPV infections are transient and cleared by the immune system within one to two years, producing no lasting lesion [6], and cervical HPV prevalence peaks in the years following sexual debut [7]. A minority persist, and persistent infection with high-risk types—particularly HPV16 and HPV18—drives the stepwise progression from low-grade squamous intraepithelial lesions to high-grade lesions (cervical intraepithelial neoplasia grade 2 or 3, CIN2 and CIN3), carcinoma in situ and, ultimately, invasive carcinoma [8]. These two genotypes account for the majority of invasive cervical cancers worldwide [9]. Because this progression unfolds over years, there is a wide window during which precancerous change can be intercepted; the central task of screening is therefore not merely to detect HPV, but to distinguish the many women with clinically inconsequential infection from the few whose lesions are destined to progress.

Several diagnostic modalities are deployed to accomplish this triage, each interrogating a different facet of the disease. Cervical cytology (the Papanicolaou test) assesses morphological change; HPV DNA testing and genotyping detect the causative agent and stratify oncogenic risk; colposcopy visualizes the transformation zone and directs biopsy; and histopathology of biopsy or excision specimens provides the definitive grading that serves as the reference standard for all other tests. Each modality, however, carries intrinsic limitations. Cytology is subject to sampling and interpretive variability, with only moderate interobserver reproducibility even among expert pathologists [10]. Adjunctive biomarkers such as p16/Ki-67 dual staining aim to identify transforming infection and improve specificity [11], whereas HPV testing is highly sensitive but comparatively non-specific for high-grade disease, and colposcopy is operator-dependent [12].

Because no single test is simultaneously sensitive and specific, contemporary practice combines them, and the clinical value of any combination hinges on how well the tests agree; structured triage algorithms have been developed precisely to manage this complexity [13]. When cytology, HPV status and histology point to the same conclusion, management is straightforward, and modern risk-based guidelines translate concordant results into clear action thresholds [14,15]. The difficult and clinically consequential situations are the discordances: a high-grade cytological interpretation not confirmed on biopsy, or, more dangerously, a normal or low-grade smear in a woman who in fact harbors CIN2+. Systematically measuring these concordances and discordances—rather than reporting each test in isolation—reveals where a screening pathway is most likely to fail and where additional triage adds the greatest safety.

The move toward HPV-based screening is grounded in a large body of comparative evidence showing that primary HPV testing detects high-grade disease more sensitively than cytology and affords greater protection against invasive cancer [16,17,18]. Genotype-resolved testing further refines this approach: large screening studies demonstrate that HPV16/18 positivity identifies the women at highest immediate risk of CIN3+ and can be used to expedite colposcopy [19], and risk-based frameworks now formalize how combinations of results map onto management [20]. Beyond the tests themselves, the probability of high-grade disease is modulated by viral and host co-factors—infection with HPV16/18, multiple concurrent genotypes, tobacco smoking, and immunosuppression have all been associated with lesion persistence and progression, while vaccination modifies risk in the opposite direction. Genotype-specific information is therefore valuable for risk stratification because longitudinal studies show different genotype-specific risks. In the present cross-sectional study, however, a single HPV result is interpreted only as a marker associated with concurrent lesion severity and cannot distinguish transient infection from persistent or integrated infection.

Against this background, the present study analyzes a consecutive series of women evaluated for cervical pathology over three consecutive years (2022–2024) in a setting where HPV vaccination coverage is very low, a context that remains common across Central and Eastern Europe despite the ongoing transition toward organized, HPV-based programs [21,22]. We set out to characterize the demographic and virological profile of HPV-positive versus HPV-negative women, to quantify the concordance and discordance between cytology, HR-HPV genotyping, colposcopy and the histological reference standard, and to identify the factors independently associated with high-grade cervical disease. In doing so, we aim to inform pragmatic, locally applicable triage strategies for a high-burden, low-coverage population.

## 2. Materials and Methods

### 2.1. Study Design and Population

We conducted a retrospective, single-center observational cohort study of women evaluated for suspected or established HPV-induced cervical pathology over three consecutive calendar years, from 2022 through 2024, at a specialized gynecologic referral service. Eligible participants had an evaluable cervix and at least cervical cytology together with HPV testing and/or colposcopic or histological assessment documented during the study interval. Records without an evaluable cervix (including prior total hysterectomy) or without any interpretable cervical diagnostic result were not eligible. Previous treatment for CIN, pregnancy, immunosuppression, prior cervical cancer, or another gynecologic malignancy were not prespecified analytic exclusions unless they prevented interpretable cervical sampling; these histories were not captured consistently enough for subgroup analysis. Cases were assembled consecutively from the referral service records. The final cohort comprised 172 women (62 in 2022, 59 in 2023, and 51 in 2024). This is a referral-enriched, high-prevalence clinical cohort rather than a population-based primary-screening sample; prevalence-dependent measures such as PPV and NPV, and any proposed triage implications, are therefore applicable only to comparable referred populations unless externally validated. HPV vaccination coverage was very low, with only three vaccinated women in the cohort.

Data were abstracted from structured clinical spreadsheets maintained by the treating team and were fully de-identified before analysis; only the internal observation number, and no direct patient identifiers, was retained. For each woman the record captured age, area of residence (urban or rural), smoking status, HPV vaccination status, menopausal status, cervicorrhagia, and the available diagnostic cascade of cytology, HPV genotyping, colposcopy, and histopathology. The study was conducted in accordance with the Declaration of Helsinki [23] and approved by the Local Ethics Committee for Scientific Research of the “Pius Brînzeu” Emergency County Clinical Hospital Timișoara, Romania (approval no. 182, date of approval 24 September 2024).

### 2.2. Cervical Cytology and HPV Testing

Cervical cytology was obtained by conventional or liquid-based sampling and reported according to the 2014 Bethesda System [24]. For analysis, cytological interpretations were grouped into the standard categories of negative for intraepithelial lesion or malignancy (NILM), atypical squamous cells of undetermined significance (ASC-US), low-grade squamous intraepithelial lesion (L-SIL), atypical squamous cells cannot exclude high-grade lesion (ASC-H), and high-grade squamous intraepithelial lesion (H-SIL). To evaluate agreement with histology, cytology was additionally dichotomized into a low-grade category (NILM, ASC-US and L-SIL) and a high-grade category (ASC-H and H-SIL), the latter representing the cytological threshold at which high-grade histological disease is clinically anticipated.

HPV testing determined the presence of high-risk genotypes, and, where a positive result was obtained, the specific genotype or combination of genotypes was recorded (for example, HPV16, HPV18, HPV31, HPV33, HPV45, HPV51, HPV52, and others). From these data we derived a binary HPV status (positive versus negative), a binary indicator of HPV16 and/or HPV18 involvement versus other high-risk types, and a count of concurrent genotypes to flag multiple infections. Because HPV16/18 carries the highest established oncogenic risk, the 16/18 versus non-16/18 contrast was prespecified as the principal genotype comparison. A single nucleic-acid result was not interpreted as evidence of persistence, viral integration, or transcriptional activity. HPV results were abstracted from routine laboratory reports. The retrospective dataset preserved genotype calls but did not preserve the assay manufacturer, platform, DNA-isolation protocol, PCR chemistry, analytical sensitivity, complete detectable-genotype panel, or laboratory quality-control metadata; these assay-dependent technical details therefore could not be reconstructed without the source laboratory records. Adjunctive p16/Ki-67 dual-stain cytology (CINtec PLUS; Roche mtm laboratories AG, Mannheim, Germany) and recorded concomitant systemic infections were abstracted as descriptive context.

### 2.3. Colposcopy, Histology and the Reference Standard

Colposcopic examination of the cervix and transformation zone was undertaken in women with abnormal cytology or a positive HPV result according to clinical judgment, and biopsy was performed when the colposcopic or overall clinical assessment warranted tissue sampling. The retrospective record captured only a binary clinical colposcopy field (abnormal/performed with biopsy versus not indicated or unremarkable) and did not preserve a standardized graded colposcopic impression. Because histological verification was itself conditional on the clinical pathway that included colposcopy, this binary field is not an independent index-test threshold and was therefore not analyzed as a diagnostic-accuracy test against the biopsy it helped trigger. Directed biopsy, endocervical curettage and, where clinically warranted, diagnostic excision (conization) were used for definitive diagnosis. Histopathology graded lesions as CIN1, CIN2, CIN3, carcinoma in situ (CIS), or invasive squamous carcinoma [25]. Histology was available for 130 of 172 women; 42 did not undergo biopsy, typically because non-invasive findings and clinical assessment did not indicate tissue sampling. Missing histology was therefore clinically informative rather than missing completely at random.

Histology was treated as the reference standard against which the performance and agreement of the non-invasive modalities were judged. For the principal analyses, histological outcome was dichotomized at the clinically decisive threshold of CIN2 or worse (CIN2+, encompassing CIN2, CIN3, CIS and invasive carcinoma) versus CIN1 or less (≤CIN1), because CIN2+ is the accepted indication for treatment. The ordinal grade was additionally retained for severity-gradient analyses. Cyto-histological concordance was defined as agreement between the dichotomized cytology (low versus high grade) and the dichotomized histology (≤CIN1 versus CIN2+); discordant cases were further classified as cytological over-call (high-grade cytology with ≤CIN1 histology) or, of greater clinical concern, cytological under-call (low-grade cytology with CIN2+ histology).

### 2.4. Statistical Analysis

Continuous variables were summarized with the median and interquartile range (IQR), and categorical variables as absolute frequencies and percentages. The age distribution departed from normality in the HPV-positive group (Shapiro–Wilk *p* < 0.001); age was therefore compared with the Mann–Whitney U test. Associations between categorical variables were tested with the Pearson chi-square test, with Fisher’s exact test applied when expected cell counts were below five. All tests were two-sided, and *p* < 0.05 was considered statistically significant.

Agreement between each non-invasive laboratory/cytology modality and histology was quantified with Cohen’s kappa and 95% confidence intervals [26]. Sensitivity, specificity, PPV, NPV and accuracy for CIN2+ were calculated only among women with histological verification and the required index test, with Wilson-score 95% confidence intervals; these are apparent complete-case performance estimates conditional on biopsy, not population-screening estimates. Missing values were not imputed. Twelve of 172 women lacked a definitive HPV result; among the 109 HPV-positive women, 7 lacked interpretable core cytology. Histology was available for 130 women, yielding analysis-specific denominators of 123 for cytology–histology, 121 for HPV-status–histology, and 93 for genotype–histology analyses. Because biopsy verification was non-random and the retrospective dataset did not capture a sufficiently complete set of individual-level determinants of verification to support a reliable inverse-probability model, inverse-probability weighting was not applied. Instead, an extreme-case verification-bias sensitivity analysis was prespecified for HPV DNA among the 39 women with known HPV status but no histology (15 HPV-positive and 24 HPV-negative). The strength of association with CIN2+ was expressed as odds ratios (ORs) with 95% confidence intervals. HPV16/18, which is structurally defined only within HPV-positive women, was retained in a prespecified HPV-positive stratified logistic analysis rather than entered jointly with the parent HPV-status variable. The main multivariable model included age, HPV status, high-grade cytology, and cervicorrhagia, and is reported as adjusted ORs. The fixed sample size represented a census of all consecutive eligible records during 2022–2024; no a priori sample-size calculation was performed, and post hoc power was not used because precision is conveyed by confidence intervals. Analyses were performed in Python 3.14.6 (Python Software Foundation, Wilmington, DE, USA) using pandas 3.0.4, SciPy 1.18.0, and statsmodels 0.14.6.

**Figure 1:**
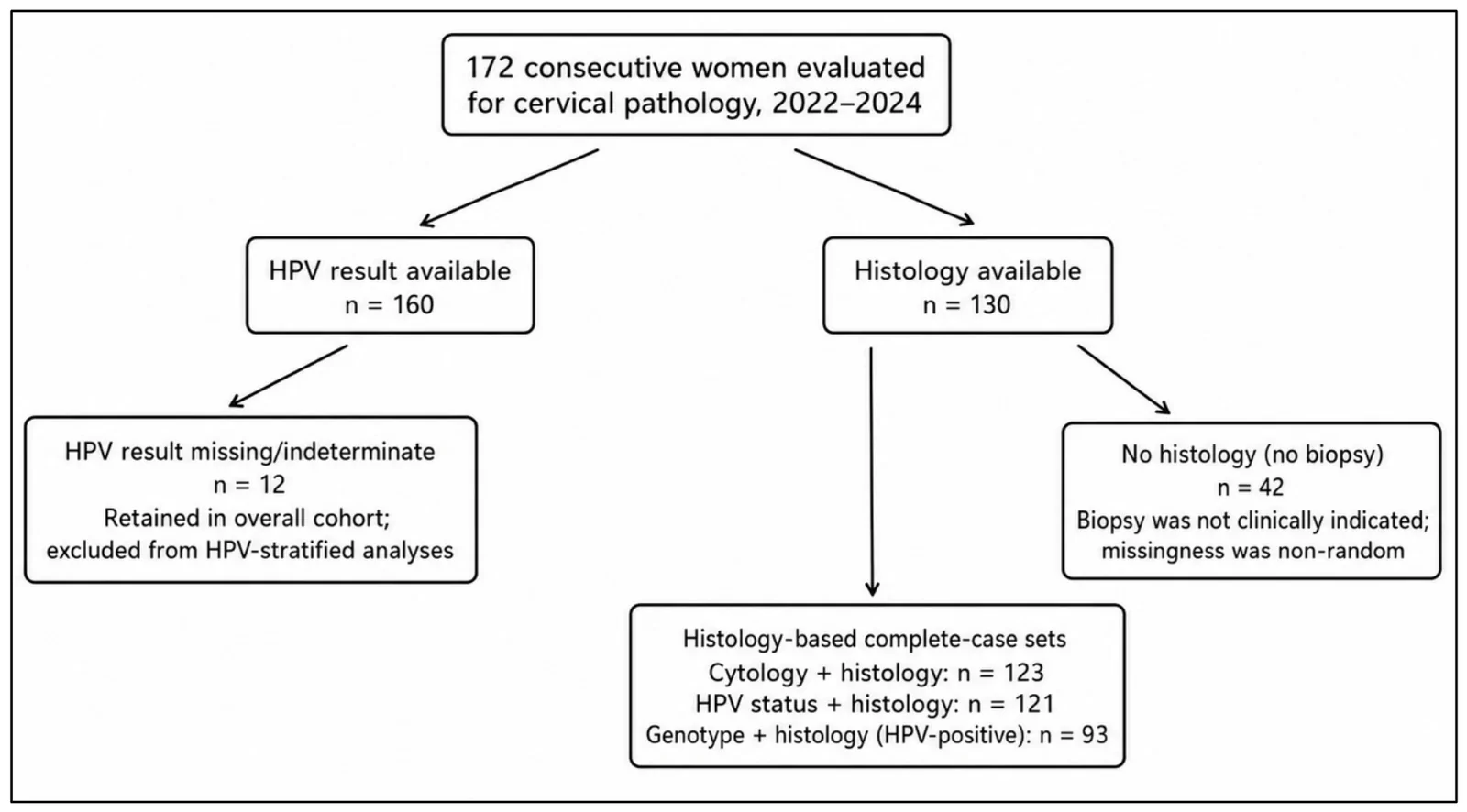
STROBE-style participant flow and analysis-specific denominators. The 172-woman referral cohort was retained for overall descriptions; analyses used complete cases for the required tests. Histology was unavailable in 42 women because biopsy was not clinically indicated, making verification non-random.

Participant disposition, missing-test patterns and analysis-specific denominators are summarized in Figure 1.

## 3. Results

The baseline demographic and clinical characteristics of the cohort, overall and stratified by HPV status, are summarized in Table 1.

The cohort comprised 172 women with a median age of 38 years (IQR 29–47) and a modest predominance of urban residents (55.2%). Only three women (1.7%) had received HPV vaccination. HPV-positive women were younger than HPV-negative women, with a median age of 33 years (IQR 28–44) versus 43 years (IQR 35–50; Mann–Whitney U, *p* < 0.001); the categorical age-group distribution also differed (*p* = 0.002). More than one-third of HPV-positive women were under 30, whereas HPV-negative women clustered in the 40–49 and ≥50 groups. Area of residence, smoking status, menopausal status, and abnormal bleeding did not differ significantly between HPV groups (all *p* > 0.09). These HPV-stratified results exclude the 12 women without a definitive HPV result, and cytology denominators vary because seven HPV-positive women lacked interpretable core cytology. The distribution of cervical cytology and histology categories according to HPV status is presented in Table 2.

The distribution of Bethesda categories differed significantly by HPV status (*p* = 0.028): high-grade cytological abnormalities (ASC-H and H-SIL) accounted for 44.1% of interpretable smears in HPV-positive women compared with only 21.6% in HPV-negative women (*p* = 0.011), while the HPV-negative group was dominated by ASC-US, the least specific abnormal category. The contrast was far more pronounced at the histological level, where the difference reached high statistical significance (*p* < 0.001). Among biopsied HPV-negative women, histology was overwhelmingly reassuring—77.8% were CIN1 and high-grade disease was rare—whereas HPV-positive women displayed the full severity spectrum, including 23 cases of CIN3, five of carcinoma in situ, and 14 invasive carcinomas. Consolidated at the decision-relevant threshold, 70.2% of biopsied HPV-positive women harbored CIN2+ compared with just 22.2% of HPV-negative women, a more than three-fold difference. This gradient establishes HPV positivity as a powerful marker of underlying high-grade disease and underlines the negative predictive value of a truly negative HPV test. At the same time, the residual 22.2% CIN2+ rate among HPV-negative biopsied women is a first signal of discordance. Dichotomized cytology is cross-classified against the histological reference standard in Table 3.

Cross-classifying dichotomized cytology against the histological reference standard in the 123 women with both tests available exposes the core concordance–discordance structure of the screening pathway. The two tests agreed in 80 of 123 cases, an overall concordance of 65.0%, but the corresponding Cohen’s kappa of 0.32 (95% CI 0.16–0.48) indicates only fair agreement once chance is accounted for—far short of the substantial agreement that would justify treating cytology as a stand-alone determinant of histological grade. The association between the two tests was nonetheless highly significant (Fisher’s exact *p* < 0.001), confirming that cytology carries real information even if it is imperfectly calibrated. High-grade cytology performed as a specific but insensitive predictor of CIN2+: specificity was 77.6% and positive predictive value 79.2%, so that a high-grade smear usually reflected genuine high-grade disease, but sensitivity was only 56.8% and negative predictive value only 54.3%. The clinical weight of the discordance lies almost entirely in one quadrant: of 43 discordant cases (35.0% of the sample), only 11 were cytological over-calls (high-grade smear, ≤CIN1 histology), whereas 32 were cytological under-calls—women with low-grade or negative cytology who in fact harbored CIN2+. The relationship between HR-HPV genotype and histological lesion severity among HPV-positive, biopsied women is detailed in Table 4.

**Figure 2:**
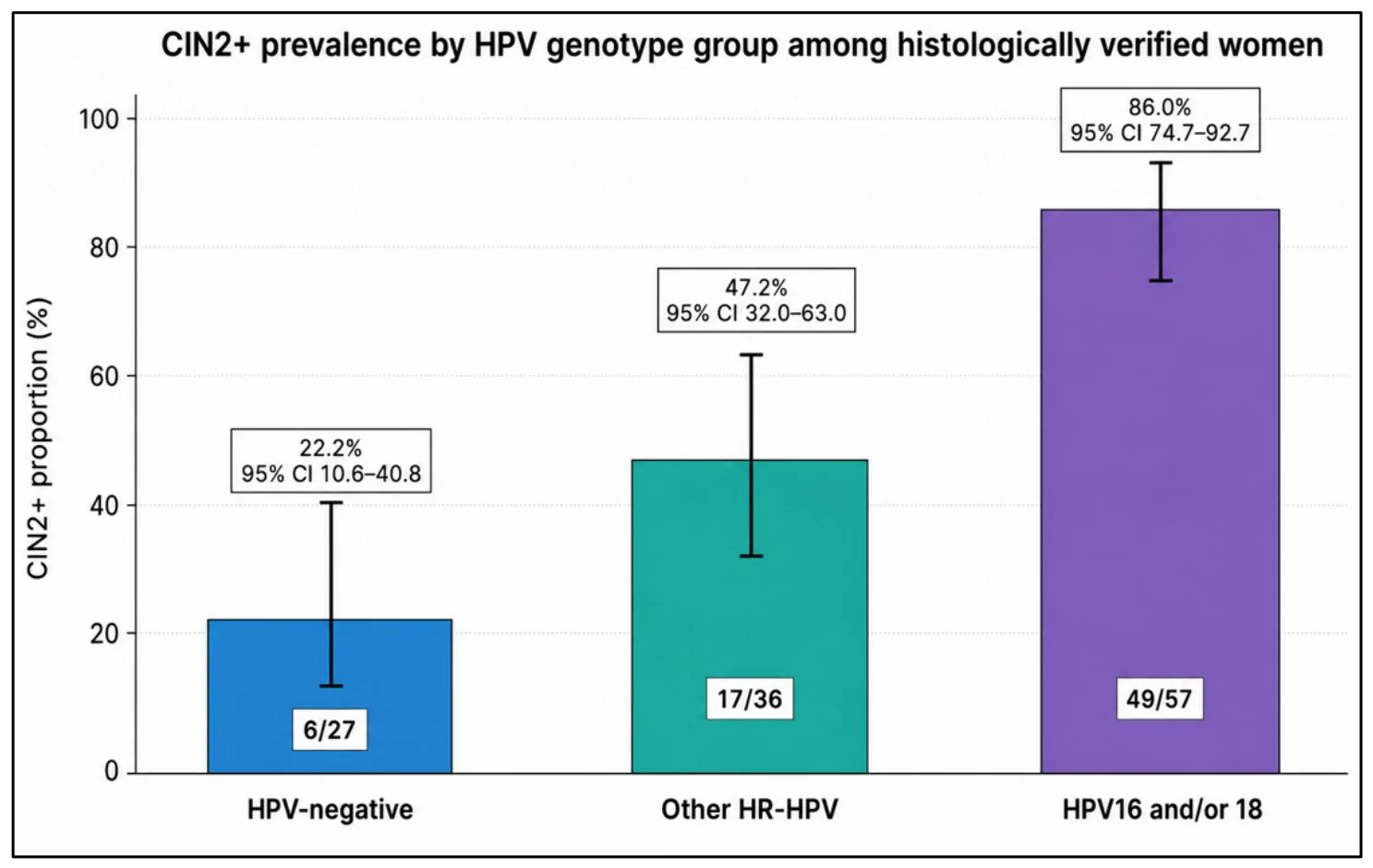
CIN2+ prevalence by HPV genotype group among histologically verified women. CIN2+ occurred in 6/27 HPV-negative women (22.2%, 95% CI 10.6–40.8), 17/36 women with non-16/18 HR-HPV (47.2%, 95% CI 32.0–63.0), and 49/57 women with HPV16 and/or HPV18 (86.0%, 95% CI 74.7–92.7). Error bars show Wilson 95% confidence intervals. Within HPV-positive women, HPV16/18 was associated with higher odds of CIN2+ than other HR-HPV types (OR 6.85, 95% CI 2.54–18.49).

HPV16 was by far the most frequent type in the cohort (57 detections), followed by HPV31, HPV33, HPV45, and HPV51, and multiple concurrent genotypes were present in 39 of 109 HPV-positive women. When genotype was dichotomized into HPV16/18 versus other high-risk types, a strong and highly significant correlation with histological severity emerged (Figure 2). Women carrying HPV16 and/or HPV18 had a CIN2+ rate of 86.0%, almost double the 47.2% observed with non-16/18 high-risk types, corresponding to an odds ratio of 6.85 (95% CI 2.54–18.49; Fisher’s exact *p* < 0.001). The relationship was monotonic across the full ordinal grade spectrum, with a Spearman correlation of 0.44 (*p* < 0.001) between HPV16/18 status and lesion grade: all five cases of carcinoma in situ and 12 of the 14 invasive carcinomas in this subgroup occurred in 16/18-positive women, whereas non-16/18 infections were concentrated in CIN1 and CIN2.

The univariable and multivariable analyses of factors associated with high-grade cervical disease (CIN2+) are reported in Table 5.

**Figure 3:**
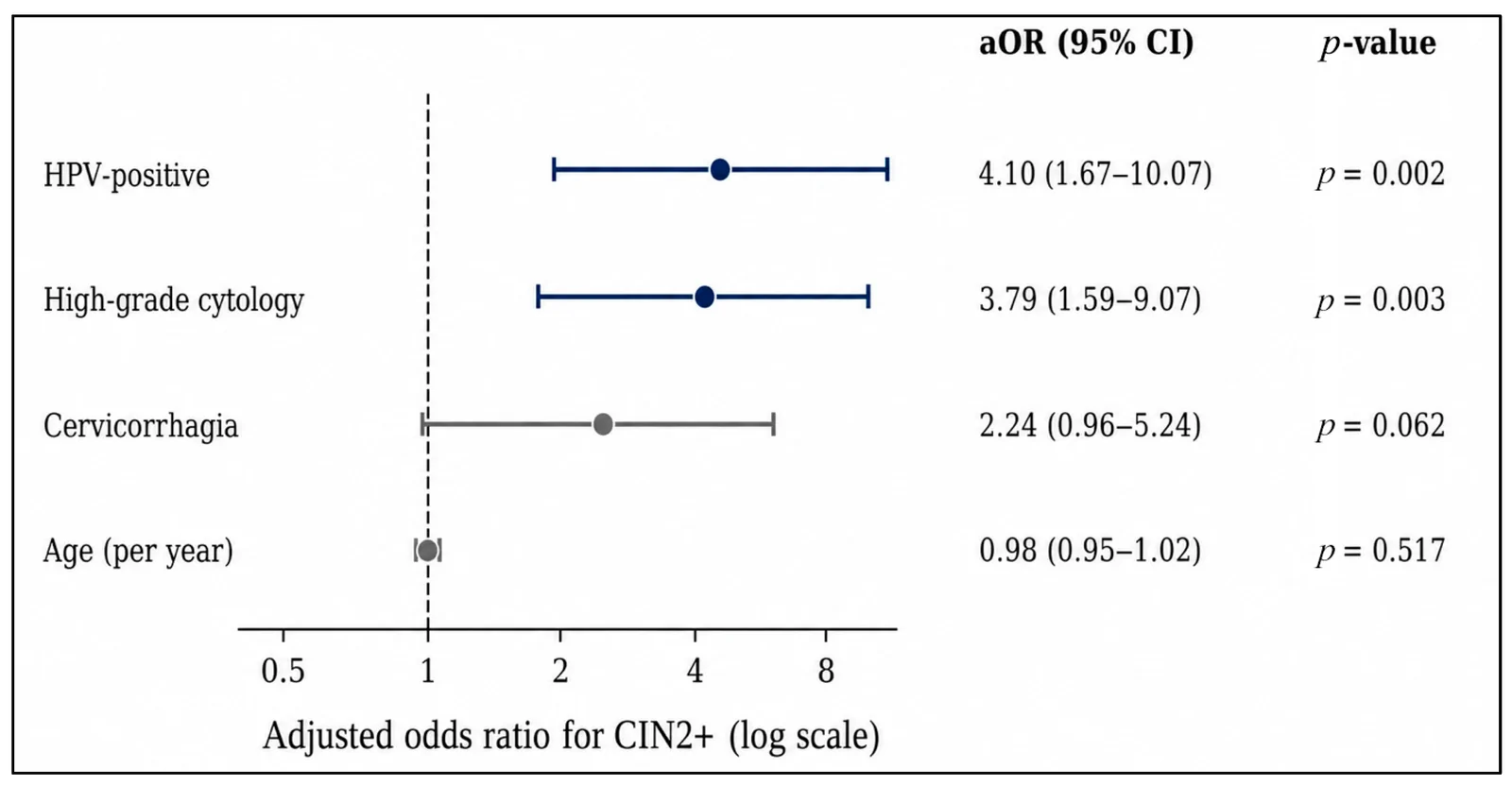
HPV status and high-grade cytology are independent predictors of CIN2+. Forest plot of adjusted odds ratios (points) with 95% confidence intervals (bars) from the multivariable logistic-regression model. HPV positivity (aOR 4.10) and high-grade cytology (aOR 3.79) remained significant after mutual adjustment, cervicorrhagia was borderline, and age had no independent effect; intervals crossing the dashed line of no effect (OR = 1) are non-significant.

Univariable analysis identified a coherent set of factors associated with CIN2+, dominated by virological and cytological markers rather than by demographic characteristics (Figure 3). The strongest single predictor was HPV positivity, which raised the odds of high-grade disease more than eight-fold (OR 8.25, 95% CI 3.01–22.63, *p* < 0.001), followed by HPV16/18 genotype within the positive subgroup (OR 6.85, *p* < 0.001) and high-grade cytology (OR 4.53, 95% CI 2.01–10.23, *p* < 0.001). Among clinical variables, abnormal bleeding was significantly associated with CIN2+ (OR 2.51, 95% CI 1.17–5.35, *p* = 0.018), consistent with symptomatic presentation reflecting more advanced lesions. Notably, several factors often assumed to increase risk were not significant here: rural residence, current smoking, and older age showed no significant univariable association, and postmenopausal status was, if anything, inversely related to CIN2+ (OR 0.40, *p* = 0.085), a pattern driven by the younger age of HPV-positive women in this referred series. When the independent contributors were assessed jointly in a multivariable logistic model, HPV positivity (adjusted OR 4.10, 95% CI 1.67–10.07, *p* = 0.002) and high-grade cytology (adjusted OR 3.79, 95% CI 1.59–9.07, *p* = 0.003) each retained strong, independent predictive value, confirming that virology and morphology capture complementary rather than redundant information. Cervicorrhagia attenuated to borderline significance after adjustment (adjusted OR 2.24, *p* = 0.062) and age exerted no independent effect.

The apparent concordance and diagnostic performance of the three analyzable non-invasive laboratory/cytology modalities within histologically verified complete cases are compared in Table 6.

**Figure 4:**
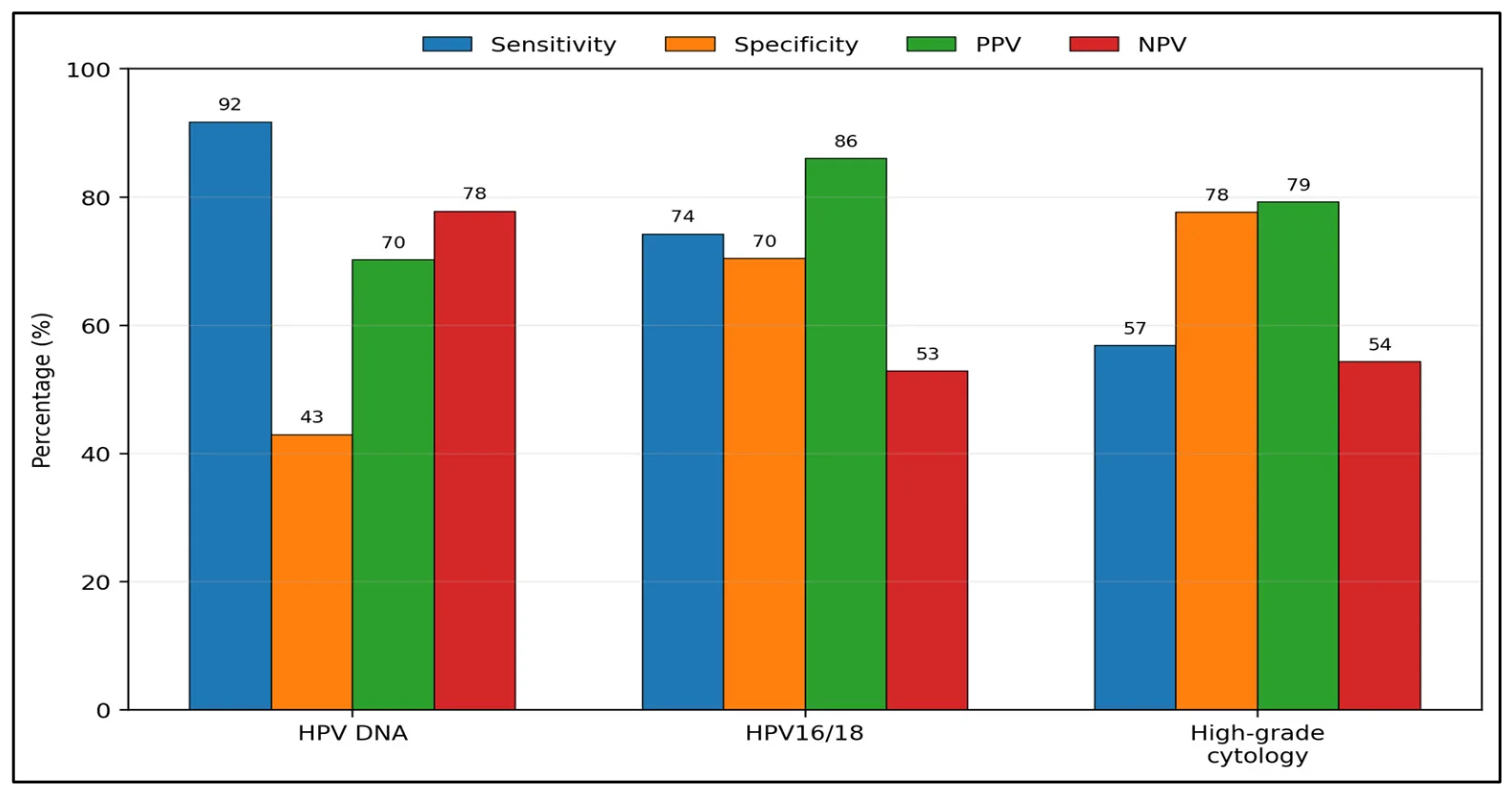
Apparent diagnostic profiles of the three analyzable non-invasive modalities among histologically verified women. Sensitivity, specificity, PPV and NPV are shown for HPV DNA positivity, HPV16/18 genotype, and high-grade cytology. Colposcopy is not plotted because the retrospective binary colposcopy variable partly determined biopsy verification and was not an independent diagnostic threshold.

Within the histologically verified subset, the three analyzable non-invasive modalities showed complementary profiles (Figure 4). HPV DNA positivity had the highest apparent sensitivity for CIN2+ (91.7%, 95% CI 83.0–96.1) but lower specificity (42.9%, 95% CI 30.0–56.7). High-grade cytology was less sensitive (56.8%, 95% CI 45.4–67.4) but more specific (77.6%, 95% CI 64.1–87.0). HPV16/18 genotyping within HPV-positive women showed the highest PPV (86.0%, 95% CI 74.7–92.7) and moderate agreement with histology (κ = 0.41). These are complete-case, verification-conditional estimates from a referral population and do not constitute population-level screening performance. The binary colposcopy field was excluded from diagnostic-accuracy benchmarking because it partly determined whether histology was obtained and did not represent an independent standardized colposcopic threshold.

As a verification-bias sensitivity analysis for HPV DNA, 39 women had a known HPV result but no histology (15 HPV-positive and 24 HPV-negative). Under the extreme best-case assignment for the HPV classifier (all 15 unverified HPV-positive women assumed CIN2+ and all 24 unverified HPV-negative women assumed ≤CIN1), sensitivity would be 93.1%, specificity 61.6%, and accuracy 78.8%. Under the opposite extreme assignment, sensitivity would be 68.8%, specificity 32.8%, and accuracy 54.4%. The width of these bounds demonstrates that selective biopsy verification can materially change apparent test performance and reinforces the need for population-based validation.

## 4. Discussion

### 4.1. Rationale, Main Findings and Contribution

This study was undertaken to characterize where cytology, HR-HPV testing, and histology agree or diverge in a real-world gynecologic referral population with very low vaccination coverage. The principal finding is not that one test is universally superior, but that the modalities capture different components of risk. HPV-positive referred women were younger and, among those biopsied, had a higher burden of CIN2+; cyto-histological agreement was only fair (κ = 0.32), with cytological under-calls outnumbering over-calls. These findings support the clinical need for layered risk assessment in referred women while also showing why single-test results should not be interpreted in isolation. Adjunctive p16/Ki-67 dual staining has been shown to improve the triage of women with abnormal cytology [27], and the limitations of colposcopy as the gatekeeper to histological diagnosis are well recognized [28]. The study is distinctive in linking cytology, genotype information and histological grade across a three-year consecutive referral series and in explicitly quantifying discordant pathways that may lead to missed high-grade disease.

Within HPV-positive, histologically verified women, HPV16/18 was strongly associated with concurrent CIN2+ (OR 6.85, 95% CI 2.54–18.49) and higher ordinal lesion grade. This genotype result was retained through a prespecified HPV-positive stratified analysis rather than being discarded because of its structural relationship with overall HPV status. In the main multivariable model, HPV positivity and high-grade cytology remained independently associated with CIN2+, supporting the view that virologic and morphologic information are complementary. The genotype finding should nevertheless be interpreted as cross-sectional risk stratification: a single DNA test cannot establish persistent infection, viral integration or transcriptional activity. The observed gradient is consistent with the established oncogenic biology of HPV16/18 [29] but does not demonstrate those longitudinal mechanisms in individual patients.

### 4.2. Study Population and Referral-Setting Implications

The source population is central to interpretation. Women were evaluated at a specialized gynecologic referral service for suspected or established HPV-related cervical pathology, yielding an HPV-positive proportion of 68.1% among those with definitive results and a high prevalence of CIN2+ among biopsied women. This spectrum is fundamentally different from an average-risk community screening population. Because PPV and NPV depend on disease prevalence, the predictive values reported here are expected to be higher and lower, respectively, than they would be in lower-prevalence primary screening. In addition, histology was obtained selectively rather than universally: women with reassuring non-invasive findings were less likely to undergo biopsy. This non-random verification can inflate apparent sensitivity and depress apparent specificity, as illustrated by the extreme-case sensitivity analysis. Accordingly, the study estimates describe a referred, histologically verified subset and should not be used as direct estimates of screening-test performance in the general population.

### 4.3. Comparison with Existing Evidence

Our results are directionally consistent with the international evidence supporting primary HPV testing as a more sensitive population-screening method than cytology and with risk-based frameworks that use HPV16/18 to identify higher-risk HPV-positive women [16,17,18,19,20]. However, the magnitude of HPV positivity in this referral cohort is far above that reported in general-population epidemiologic studies and organized screening settings [7,22], emphasizing spectrum effects rather than a contradiction in test biology. Central and Eastern European programs also differ substantially in vaccination uptake, screening organization, and follow-up capacity [21,22]; these contextual differences can change the balance between sensitivity, specificity, and referral burden. In well-organized high-income screening systems, primary HPV testing is paired with validated assays, structured recall, and risk-based triage, whereas opportunistic or fragmented systems face greater risk of both loss to follow-up and over-referral. The present findings therefore add local referral-setting evidence to, rather than replace, the broader population-screening literature.

### 4.4. Strengths and Limitations

Strengths include the consecutive three-year clinical series, linkage of cytology and genotype data with a histological reference standard, use of the clinically relevant CIN2+ threshold, and explicit reporting of discordance and analysis-specific denominators. Several limitations materially constrain inference. First, the study is single-center and referral-enriched, so prevalence-dependent predictive values are not generalizable to primary screening. Second, histology was missing non-randomly in 42 women because biopsy depended on prior clinical findings; complete-case sensitivity and specificity are therefore vulnerable to partial verification bias, and individual-level data were insufficient for a defensible inverse-probability weighting model. Third, the retrospective dataset did not preserve manufacturer/platform, extraction, PCR, analytical-sensitivity, full genotype-panel, or laboratory quality-control metadata, preventing assay-specific performance assessment. Fourth, the binary colposcopy variable did not preserve a standardized graded impression and could not be evaluated independently from the biopsy pathway it helped trigger. Fifth, a single HPV measurement cannot distinguish transient from persistent infection, and longitudinal clearance or persistence was not assessed. Finally, the fixed cohort was a census of eligible records rather than a prospectively powered sample, some clinical histories such as immunosuppression or previous CIN treatment were incompletely captured, and no external validation cohort was available.

### 4.5. Clinical Implications and Context-Sensitive Screening Strategy

At the population level, the strongest evidence supports primary HR-HPV screening when validated assays, organized recall, and appropriate triage are available [16,17,18,19,20]. The present referral-cohort data do not establish a new population screening algorithm. For clinical pathways resembling our high-risk referral setting, they support a layered approach in which HR-HPV testing is followed by partial HPV16/18 genotyping, with cytology and, where available, dual-stain testing used to refine intermediate risk before colposcopy-directed biopsy. Local implementation should account for baseline HPV and CIN2+ prevalence, age distribution, vaccination coverage, previous screening history, immunosuppression, laboratory capacity, colposcopy access, and the likelihood of follow-up. Vaccination remains essential primary prevention and should be expanded alongside screening [30].

Clinical translation requires particular caution. If thresholds derived from this high-prevalence referral cohort were copied directly into a low-prevalence community screening program, the same sensitive strategy could generate substantially more false-positive referrals and unnecessary colposcopy because PPV would fall as underlying disease prevalence decreases. Conversely, raising thresholds to reduce referral burden could miss disease in populations with poor follow-up. Population implementation should therefore use externally validated risk thresholds and quality-assured HPV assays, with prospective monitoring of positivity rates, colposcopy referrals, CIN2+/CIN3+ yield and harms. Our findings are best viewed as evidence for complementarity of tests within a referred population, not as a stand-alone justification for broad screening policy.

## 5. Conclusions

In this three-year, specialized gynecologic referral cohort with very low HPV vaccination coverage, cytology and HR-HPV testing showed complementary but imperfect agreement with histology. HPV positivity was strongly associated with CIN2+, cyto-histological agreement was only fair, and HPV16/18 identified a high-risk subgroup among HPV-positive, biopsied women. However, diagnostic-performance estimates were calculated in selectively histologically verified complete cases and are susceptible to verification and spectrum bias. These data therefore support layered risk assessment in comparable referral settings—HR-HPV testing with genotype- and cytology-based refinement followed by colposcopy-directed biopsy when clinically indicated—but should not be extrapolated directly to average-risk population screening without external validation and locally calibrated referral thresholds.

## Figures and Tables

**Table 1:** Baseline demographic and clinical characteristics of the cohort, overall and by HPV status.

Characteristic	Overall (*n* = 172)	HPV-Positive (*n* = 109)	HPV-Negative (*n* = 51)	*p*-Value
Age, years—median (IQR)	38 (29–47)	33 (28–44)	43 (35–50)	<0.001 ‡
Age group < 30	46 (26.7%)	38 (34.9%)	6 (11.8%)	0.002 §
Age group 30–39	47 (27.3%)	33 (30.3%)	12 (23.5%)	
Age group 40–49	49 (28.5%)	26 (23.9%)	19 (37.3%)	
Age group ≥ 50	30 (17.4%)	12 (11.0%)	14 (27.5%)	
Urban residence	95 (55.2%)	64 (58.7%)	25 (49.0%)	0.327 §
Current smoker	17/113 (15.0%)	10/85 (11.8%)	7/28 (25.0%)	0.125 ¶
HPV-vaccinated	3 (1.7%)	3 (2.8%)	0 (0%)	0.552 ¶
Postmenopausal	29 (16.9%)	13 (11.9%)	12 (23.5%)	0.099 §
Cervicorrhagia (bleeding)	68/171 (39.8%)	42 (38.5%)	19 (37.3%)	0.982 §

IQR, interquartile range. Twelve women lacked a definitive HPV result and are included only in the overall column. ‡ Mann–Whitney U test; § Pearson chi-square; ¶ Fisher’s exact test.

**Table 2:** Distribution of cervical cytology and histology categories according to HPV status.

Category	HPV-Positive	HPV-Negative	*p*-Value
Cytology (Bethesda)			0.028 §
NILM	3 (2.9%)	0 (0%)	
ASC-US	27 (26.5%)	24 (47.1%)	
L-SIL	27 (26.5%)	16 (31.4%)	
ASC-H	17 (16.7%)	4 (7.8%)	
H-SIL	28 (27.5%)	7 (13.7%)	
High-grade cytology (ASC-H/H-SIL)	45/102 (44.1%)	11/51 (21.6%)	0.011 §
Histology (worst grade)			<0.001 §
CIN1	28 (29.8%)	21 (77.8%)	
CIN2	24 (25.5%)	4 (14.8%)	
CIN3	23 (24.5%)	1 (3.7%)	
CIS	5 (5.3%)	0 (0%)	
Invasive carcinoma	14 (14.9%)	1 (3.7%)	
CIN2+ (of those biopsied)	66/94 (70.2%)	6/27 (22.2%)	<0.001 §

Percentages for cytology and histology are column proportions among women with the respective test available. § Pearson chi-square test.

**Table 3:** Cyto-histological concordance: dichotomized cytology cross-classified against the histological reference standard (*n* = 123).

Cytology Grade	≤CIN1 Histology	CIN2+ Histology	Total
Low grade (NILM/ASC-US/L-SIL)	38 (TN)	32 (FN)	70
High grade (ASC-H/H-SIL)	11 (FP)	42 (TP)	53
Total	49	74	123

Reference standard = histology. TP, true positive; FP, false positive; FN, false negative; TN, true negative. Overall concordance 65.0%; Cohen’s κ = 0.32 (95% CI 0.16–0.48, fair agreement). Sensitivity of high-grade cytology for CIN2+ 56.8% (95% CI 45.4–67.4); specificity 77.6% (95% CI 64.1–87.0); PPV 79.2% (95% CI 66.5–88.0); NPV 54.3% (95% CI 42.7–65.4). Fisher’s exact *p* < 0.001. Estimates are conditional on histological verification.

**Table 4:** Correlation between HR-HPV genotype and histological lesion severity among HPV-positive, biopsied women (*n* = 93).

Genotype Group	≤CIN1	CIN2+	CIN2+ Rate	OR (95% CI)	*p*-Value
Other HR-HPV (non-16/18)	19	17	47.2%	1 (ref)	—
HPV16 and/or HPV18	8	49	86.0%	6.85 (2.54–18.49)	<0.001 ¶
Severity gradient by genotype				ρ = 0.44	<0.001 ‖

Full grade distribution—Other HR-HPV: CIN1 19, CIN2 9, CIN3 6, CIS 0, invasive 2; HPV16/18: CIN1 8, CIN2 15, CIN3 17, CIS 5, invasive 12. OR, odds ratio for CIN2+. —, not applicable (reference category). ¶ Fisher’s exact test; ‖ Spearman rank correlation between HPV16/18 status and ordinal histological grade.

**Table 5:** Univariable and multivariable analysis of factors associated with high-grade cervical disease (CIN2+).

Factor	CIN2+/Total	Unadjusted OR (95% CI)	*p*-Value	Adjusted OR (95% CI)
Age ≥ 50 vs. <30	10/21 vs. 24/34	0.38 (0.12–1.17)	0.152 ¶	0.98 per year (0.95–1.02)
Rural vs. urban residence	36/57 vs. 44/73	1.13 (0.55–2.31)	0.856 ¶	not retained
Current smoker vs. non-smoker	3/10 vs. 48/88	0.36 (0.09–1.47)	0.188 ¶	not retained
Postmenopausal vs. premenopausal	9/21 vs. 71/109	0.40 (0.16–1.04)	0.085 ¶	not retained
Cervicorrhagia vs. none	39/53 vs. 40/76	2.51 (1.17–5.35)	0.018 ¶	2.24 (0.96–5.24), *p* = 0.062
HPV-positive vs. negative	66/94 vs. 6/27	8.25 (3.01–22.63)	<0.001 ¶	4.10 (1.67–10.07), *p* = 0.002
HPV16/18 vs. other HR-HPV	49/57 vs. 17/36	6.85 (2.54–18.49)	<0.001 ¶	—
High-grade vs. low-grade cytology	42/53 vs. 32/70	4.53 (2.01–10.23)	<0.001 ¶	3.79 (1.59–9.07), *p* = 0.003

OR, odds ratio; CI, confidence interval. —, not applicable (not entered in the main multivariable model; see below). The main multivariable model included age, HPV status, high-grade cytology, and cervicorrhagia. ¶ Fisher’s exact test for univariable comparisons. HPV16/18 was not discarded from biological analysis: because genotype is defined within HPV-positive women, it was evaluated in the prespecified HPV-positive stratified analysis (Table 4) rather than entered jointly with the parent HPV-status variable.

**Table 6:** Apparent diagnostic performance of non-invasive laboratory/cytology modalities among histologically verified complete cases (detection of CIN2+).

Modality	*n*	Sens, % (95% CI)	Spec, % (95% CI)	PPV, % (95% CI)	NPV, % (95% CI)	Accuracy, % (95% CI)	Cohen’s κ (95% CI)
HPV DNA positivity	121	91.7 (83.0–96.1)	42.9 (30.0–56.7)	70.2 (60.3–78.5)	77.8 (59.2–89.4)	71.9 (63.3–79.1)	0.37 (0.19–0.55)
HPV16/18 genotype	93	74.2 (62.6–83.3)	70.4 (51.5–84.1)	86.0 (74.7–92.7)	52.8 (37.0–68.0)	73.1 (63.3–81.1)	0.41 (0.21–0.61)
High-grade cytology	123	56.8 (45.4–67.4)	77.6 (64.1–87.0)	79.2 (66.5–88.0)	54.3 (42.7–65.4)	65.0 (56.3–72.9)	0.32 (0.16–0.48)

Sens, sensitivity; Spec, specificity; PPV/NPV, positive/negative predictive value. All proportions include Wilson 95% confidence intervals. These estimates are conditional on biopsy verification and should not be interpreted as population-screening sensitivity, specificity or predictive values. κ interpreted per Landis–Koch: fair 0.21–0.40, moderate 0.41–0.60.

## Data Availability

The anonymized data supporting the findings of this study are available from the corresponding author on reasonable request, subject to institutional data-sharing agreements.
